# Antinociceptive Effects of AGAP, a Recombinant Neurotoxic Polypeptide: Possible Involvement of the Tetrodotoxin-Resistant Sodium Channels in Small Dorsal Root Ganglia Neurons

**DOI:** 10.3389/fphar.2016.00496

**Published:** 2016-12-20

**Authors:** Chun-Li Li, Xi-Fang Liu, Gui-Xia Li, Meng-qi Ban, Jian-Zhao Chen, Yong Cui, Jing-Hai Zhang, Chun-Fu Wu

**Affiliations:** ^1^Department of Pharmacology, Shenyang Pharmaceutical UniversityShenyang, China; ^2^Department of Biochemistry, Shenyang Pharmaceutical UniversityShenyang, China

**Keywords:** Voltage-gated sodium channels, Nav1.8, Nav1.9, pain, scorpion toxins

## Abstract

Antitumor-analgesic peptide (AGAP) is a novel recombinant polypeptide. The primary study showed that AGAP 1.0 mg/kg exhibited strong analgesic and antitumor effects. The tail vein administration of AGAP potently reduced pain behaviors in mice induced by intraplantar injection of formalin or intraperitoneal injection of acetic acid, without affecting basal pain perception. To further assess the mechanisms of AGAP, the effects of AGAP on sodium channels were assessed using the whole-cell patch clamp recordings in dorsal root ganglia (DRG) neurons. The results showed that AGAP (3–1000 nM) inhibited the sodium currents in small-diameter DRG neurons in a dose-dependent manner. 1000 nM AGAP could inhibit the current density-voltage relationship curve of sodium channels in a voltage-dependent manner and negatively shift the activation curves. 1000 nM AGAP could reduce the tetrodotoxin-resistant (TTX-R) sodium currents by 42.8% in small-diameter DRG neurons. Further analysis revealed that AGAP potently inhibited Na_V_1.8 currents by 59.4%, and negatively shifted the activation and inactivation kinetics. 1000 nM AGAP also reduced the Na_V_1.9 currents by 33.7%, but had no significant effect on activation and inactivation kinetics. Thus, our results demonstrated that submicromolar concentrations of AGAP inhibited TTX-R sodium channel in rat small-diameter DRG neurons. It is concluded that these new results may better explain, at least in part, the analgesic properties of this polypeptide.

## Introduction

Asian scorpion *Buthus martensii* Karsch, widely distributed in Mongolia, Korea, and China, has been one of the indispensable materials used in Chinese traditional medicine for thousands of years. In China, scorpions and their tissues have been extensively used for treatment of analgesia, inflammatory, epilepsy, convulsion, anticancer, and so on. It is well known that scorpion venom is complex mixture substances. It is a particularly rich source interacting specifically with various ion channels in excitable cell membranes. Proteins from Scorpion venom modes of action fall within two major categories: one is pore-blocking proteins which block the movement of ions and the other alters the conformation of ion channel and also the statement of ion channel, such as open, closed or activation (Lai et al., 2004).

Scorpion polypeptides have long chain and short chain peptides. In 2003, the expression and purification of a novel antitumor-analgesic peptide (AGAP) were reported in *E. coli* (Herzog et al., 2001). BmK AGAP is a typical long-chain scorpion toxin, which is composed of 66 amino acids cross-linked by four disulfide bridges (Cys12–Cys63, Cys16–Cys36, Cys22–Cys46, and Cys26-Cys48). The gene cloning (GenBank No. AF464898), protein expression, and characterization of the encoding gene of this peptide have been investigated (Liu et al., 2003). The primary study showed that AGAP exhibited strong analgesic and antitumor effects, but the analgesic mechanisms remain unclear in VGSCs (Liu et al., 2003).

Voltage-gated sodium channels mediate the inward sodium current and are critical for the initiation and propagation of action potentials in excitable tissues, including the brain and peripheral nerves (Payandeh et al., 2011). Nav1.3, Nav1.7, Nav1.8, and Nav1.9 have been identified as possible targets for analgesics. The development of chronic pain can be caused to peripheral nerve injury which is related to hyperexcitability of sensory neurons in DRG (Dib-Hajj et al., 2010; Ebersberger et al., 2011; Djouhri et al., 2012). At least two types of sodium currents can be recorded in small-diameter neuron of rat DRG (Caffrey et al., 1992).

Previous reports have demonstrated that TTX-R is a unique expression in the DRG neurons. Electrophysiological studies in sodium channels also have confirmed that TTX-R sodium currents may play a key role in setting the action potential conduction in the unmyelinated C fibers which arise from small DRG neurons (Jeftinija, 1994; Quasthoff et al., 1995). TTX-R sodium channels, Nav1.8 and Nav1.9, both are strongly implicated in the molecular mechanisms of nociception. In contrast to fast activating and the rapidly inactivating TTX-S channels, Nav1.8 channels exhibit ∼10-fold slower kinetics of activation and inactivation, whereas Nav1.9-mediated currents are persistent current with a more hyperpolarized voltage dependence and ultraslow recovery from inactivation (Cummins et al., 1999).

Many kinds of long-chain neurotoxins which modulate sodium channels and calcium channels have been purified from scorpion venom (Valdivia et al., 1992; Tan et al., 2001). Our previous work has shown that AGAP potently inhibited voltage gated calcium channels (VGCCs), especially high-voltage activated calcium currents in rat DRG neurons (Liu et al., 2014). Here, we examined whether AGAP could attenuate pain behaviors in mice, and further assess the analgesic mechanisms of AGAP, the present study was therefore designed to evaluate the mechanisms of recombinant AGAP on the TTX-R sodium channels in rat small-diameter DRG neurons.

## Materials and Methods

### Animals

Adult 6 weeks male Sprague Dawley rats (160–220 g), and Kunming mice (18–22 g) were used for the experiments. The animals were housed at room temperature (23–26°C) under a 12-h light/dark cycle with *ad libitum* access to food and water. All procedures were carried out in accordance with the Committee Ethics of Animal Experiments of the Shenyang Pharmaceutical University.

### Behavioral Analysis

#### Acetic Acid-Induced Writhing Test

The male mice were treated with AGAP (0.25, 0.5, 1.0 mg/kg), the reference antinociceptive drug morphine (2 mg/kg), or saline as a negative control (*n* = 12 per group). The methods included randomization and blinding in all vivo experiments. Acetic acid (0.8% v/v, 10 ml/kg) was injected intraperitoneally, and the mice were placed in a plastic cage. The abdominal writhes were defined as extensions of the abdomen with outstretching of the hind limbs. The intensity of pain was quantified by counting the number of writhes occurring for 30 min after acetic acid injection.

#### Hot Plate Test

The hot plate was an electrically heated surface kept at a constant temperature of 55.0 ± 0.5 °C. Female mice (*n* = 12 per group) were placed on the heated surface, and the latency of pain (jumping or licking of the paws) was recorded at 0, 30, 60, 90, and 120 min after drug administration, whereupon the reaction time of 0 min is the start of the test. As previous researches indicated, 2 mg/kg morphine offered an ideal analgesic effect in the visceral pain test (Meymandi and Keyhanfar, 2013), while in the hot plate test, morphine couldn’t elicit significant analgesic effects at low dosages until we raised the dosage to 5 mg/kg (Casarrubea et al., 2012; Araújo et al., 2016). Morphine (5.0 mg/kg) was used as the reference drug. Antinociceptive effects of AGAP and morphine were compared in dose-response and time course experiments. A cut-off time of 60 s was chosen to indicate complete analgesia in order to avoid potential harmful effects. The latencies of pain were recorded for each animal (Yamamoto et al., 2002).

#### Formalin Test

Formalin test was performed in male mice as Rosa et al. (2008) described. Formalin solutions were prepared at 5% in saline from a formalin stock and injected intraplantarly to the left hind paw in a volume of 20 μl. AGAP at concentrations ranging from 0.25 to 1.0 mg/kg was administered intravenously 20 min before formalin injection. Morphine was administered at a dose of 2 mg/kg (*n* = 12 per group). 30 min before formalin injection. After the formalin injection, mice were immediately placed in a clear, transparent box (11 cm × 11 cm × 16 cm) with a mirror placed underneath at a 45° angle to view the animals’ paws completely. Then, mice were observed for 30 min after the formalin injection, the times of licking or flinching the injected hind paw from 0 to 5 min (first phase) and from 15 to 30 min (second phase) was recorded as indicative of nociception.

#### Rotarod Test

Fine motor coordination and learning was assessed by using the rotarod test (Munro, 2009). A control (saline) group and AGAP group (treated 1.0 mg/kg; *n* = 10) were utilized. The fall-off latency to the first fall was recorded.

### Immunohistochemistry

Rats were deeply anesthetized, and the L_4-6_ lumbar DRG were removed quickly from the spinal cord, fixed with 4% paraformaldehyde in 0.1 M phosphate buffer overnight at 4°C. After the tissues embedded by paraffin, a series of 5 μm thick sections were cut for immunohistochemistry. Primary antibodies targeting rat Na_V_1.8 (1:200; Alomone), Na_V_1.9 (1:200; Alomone) and NF200 (1:200; Sigma) were performed for immunohistochemistry overnight at 4°C. After washing three times with PBST, the sections were incubated with secondary antibodies (1:100; sigma) for 1 h at 37°C. Fluorescent images were captured in an Olympus BX40 microscope (Olympus, Tokyo, Japan).

### Preparation of DRG Neurons

#### Primary Neuronal Cultures

L_4-6_ DRG tissue was removed from day 1–3 rat pups in Ca^2+^ and Mg^2+^ free Hank’s, minced, and incubated for 15 min at 37°C in enzyme solutions containing trypsin and collagenase. The tissue was triturated in culture medium containing 1:1 DMEM/F-12, 10% fetal calf serum, 5% horse serum, 1.5 mg/ml bovine serum albumin, 100 U/ml penicillin and 0.1 mg/ml streptomycin and plated (600–800 cells/mm^2^) on glass coverslips coated with Poly-L-Lysine. The cells were maintained at 37°C in a humidified 95% air/5% CO_2_ incubator overnight, and studied with whole-cell patch-clamp techniques after short-term culture (12–24 h).

#### DRG Neurons Acutely Isolated

Preparation of DRG neurons was established as described previously (Yu et al., 2011). Briefly, SD rats weighing about 150 g (6 weeks of age) were deeply anesthetized, L_4-6_ DRG neurons were dissociated using enzyme digestion as previously described with slight modifications. The cells were then triturated and plated onto acid-washed coverslips that had been coated previously with 0.01% poly-L-lysine. Media consisted of Dulbecco’s Modified Eagle Medium/Ham’s F12 medium (DMEM/F12) supplemented with 10% (v/v) heat-inactivated horse serum and 10% (v/v) fetal bovine serum. Cell densities at plating were 800–1000 cells/ml. The cells were maintained at 37°C in a humidified 95% air/5% CO_2_ incubator and were studied between 2–12 h after removal from the animal.

### Electrophysiological Recordings

Voltage-gated sodium currents of L_4-6_ DRG neurons were recorded in the whole-cell configuration of the patch clamp method (Li et al., 2010). During experiments, membrane potential and currents were recorded from small-diameter DRG neurons using the whole-cell patch clamp technique under visual control with Nikon TE2000-U inverted microscope. Patch-clamp electrodes were pulled with a P-97 puller, and had a resistance of 3–5 MΩ. The experiment of whole-cell patch clamp was carried out using an AxoPatch 200B amplifier (Axon Instruments, Foster City, CA, USA); all signals were low pass filtered at 1.5 kHz and the sampling rate was 10 kHz, then sampled data were stored digitally on a computer for further analysis. The pipette solution contained (in mM): 100 CsCl, 30 CsF, 8 NaCl, 2.4 CaCl_2_, 1 MgCl_2_, 5 EGTA, 4 Na_2_ATP, 10 HEPES, 0.4 GTP (pH 7.3, Osmolarity is 290–300 mOsm). The bathing solution contained (in mM): 100 choline chloride, 40 NaCl, 3 KCl, 2.5CaCl_2_, 1 MgCl_2_, 10 HEPES, 10 glucose, 0.005 LaCl_3_ (pH 7.4, Osmolarity is 290–300 mOsm), supplemented with 1 μM TTX when the currents of TTX-R, Na_V_1.8 and Na_V_1.9 channel were recorded. The pipette offset was used to back the liquid junction potential to zero before patch formation. Capacitive transients and series resistances were compensated electronically by 70–80%. After seal formation and membrane rupturing, the cells were allowed to stabilize for 3 min before rupturing the pulse protocols. All experiments were carried out at room temperature (23 ± 2°C).

### Reagents

CsCl, CsF, TTX, LaCl_3_, HEPES, Poly-L-lysine, EGTA, Na_2_ATP, and GTP were purchased from Sigma-Aldrich, whereas DMEM/F12, horse serum, and fetal bovine serum were from Gibco. Penicillin and streptomycin were purchased from Invitrogen. AGAP was synthesized by the laboratory of Dr. Zhang Jinghai.

Stock solutions of AGAP were prepared in physiological saline and diluted in assay buffer immediately before use. AGAP was dissolved in the bath solution. Throughout the experiment, bath or AGAP solutions were delivered to the DRG neurons via a fast gravity-driven perfusion system. The doses of AGAP were determined by the preliminary experiments *in vivo* or *vitro* experiment.

### Data Analysis

#### The Dose-Response Curves

The dose-response curves for AGAP was determined from a curve fit of the Hill equation to the data points: *f* = 1 / {1 + (*IC*_50_ / [*AGAP*])^nH^}, where *f* is the fractional current block at the test potential, nH is the Hill coefficient, and [*AGAP*] is the concentration of AGAP. The inhibition effect calculated as the mean reduction of maximum peak amplitude.

#### Establish Voltage-Dependent Steady-State Activation Curves

The voltage dependence of activation was determined using standard protocols. The conductance *G* (*V)* was calculated according to *G = I* / (*V - V*_Na_), where *V*_Na_ is the reversal potential, *V* is the test pulse potential and *I* is the current amplitude. Normalized peak conductance was fitted by the following Boltzmann equation: *G* / *G*_max_ = 1 / {1 + exp[(*V*_1/2_ - *V*) / *k*]}, where *G*_max_ is the maximum conductance, *V*_1/2_ is the membrane potential of half-maximal activation and *k* is the slope factor.

#### Establish Voltage-Dependent Steady-State Inactivation Curves

Steady state inactivation curves were determined with a Boltzmann fit of the data using: *I* / *I*_max_ = 1 / {1 + exp[(*V* - *V*_1/2_) / *k*]}, where *I* is the current amplitude, *I*_max_ is the maximal current amplitude, *V* is the prepulse, *V*_1/2_ is the prepulse voltage at which the current amplitude is half maximum, and *k* is the slope factor.

The voltage-clamp data were digitized and analyzed using pClamp 10.0 software (Axon Instruments). Multiple comparisons were evaluated by one-way ANOVA followed by Tukey’s test, two-way ANOVA or repeated-measure two-way ANOVA followed by Bonferroni test. When only two groups were compared, an unpaired *t*-test was used. Data were expressed as mean ± standard error of mean (SEM). *P* < 0.05 indicated statistically significant differences.

## Results

### Effects of AGAP on Acetic Acid-Induced Abdominal Writhing Visceral Pain

**FIGURE 1:**
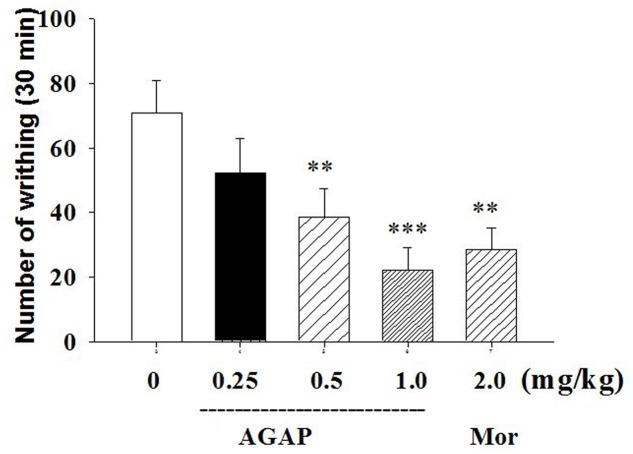
**Effect of Antitumor-analgesic peptide (AGAP) on acetic acid-induced writhing test in mice**. After intravenously injection of AGAP, mice received an intraperitoneal injection of 0.8% acetic acid, and abdominal writhes were counted for 30 min. AGAP dose-dependently inhibited the total number of writhes. ^∗∗^*p* < 0.01, ^∗∗∗^*p* < 0.001 compared with control group. It is no significance of comparison between morphine and 1.0 mg/kg AGAP (*p* > 0.05). Data are shown as the mean ± SEM (*n* = 12 per group). *P* values are from one-way ANOVA followed by Tukey’s test.

We first tested the effects of intravenously administered AGAP to reduce pain-related behaviors after intraperitoneal administration of 0.8% acetic acid. As shown in **Figure 1**, intraperitoneal administration of acetic acid induced significant abdominal writhing in mice. When mice were pretreated with AGAP, we observed significant dose-dependent effect of AGAP (0.5–1.0 mg/kg) on acetic acid-induced writhing relative to vehicle-treated mice. In order to compare the antinociceptive effect of AGAP, a group of mice were administered with morphine. The results showed that 2.0 mg/kg morphine also significantly reduced the total number of writhes. Inhibition ratio of writhes is 69.8 and 60.2% after administration of AGAP (1.0 mg/kg) and morphine (2.0 mg/kg), respectively (*P* < 0.001 and *P* < 0.01, respectively, *n* = 12 in each group).

### Effects of AGAP on the Hot Plate-Induced Nociception

**FIGURE 2:**
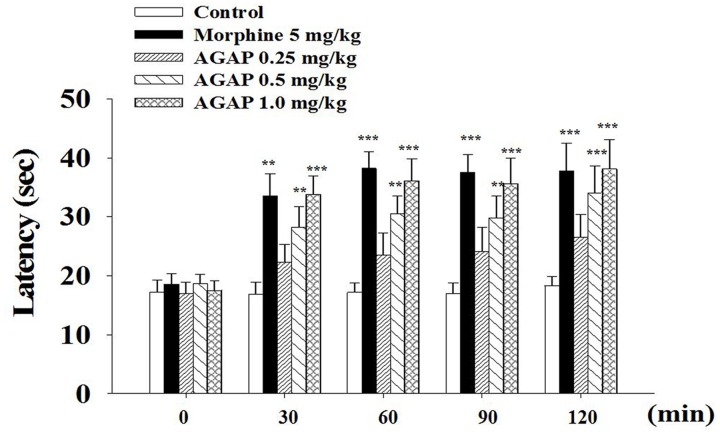
**Reaction time of animals in hot plate test: 0, 30, 60, 90, and 120 min after treatment of AGAP and morphine**. Animals were pretreated with vehicle, morphine (5 mg/kg), AGAP (0.25, 0.5, and 1.0 mg/kg) prior to the tests at 55°C. Each column represents the mean with X ± SEM. for twelve mice in each group. The symbols denote the significance levels: ^∗∗^*p* < 0.01, ^∗∗∗^*p* < 0.001 compared with control group. Morphine and 1.0 mg/kg AGAP were not statistical difference (*p* > 0.05). Data are shown as the mean ± SEM (*n* = 12 per group). *P* values are from two-way ANOVA followed by Bonferroni test.

To complement and extend our *in vivo* studies, the antinociceptive effects of AGAP were studied in the hot plate test in mice. AGAP was injected into the caudal vein of mice, the physiological saline was a control, and the pain latency was recorded. On the hot plate assay, AGAP (0.5–1.0 mg/kg) significantly increased the response latency compared with the control group (**Figure 2**). The observed pharmacological action was similar to morphine (5.0 mg/kg). The latency did not return to baseline within 120 min for test.

### Effects of AGAP on Formalin-Induced Nociception

**FIGURE 3:**
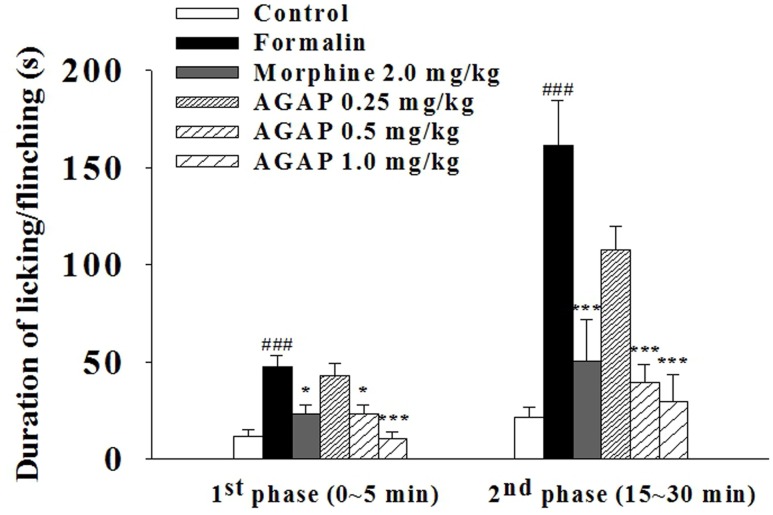
**Effect of AGAP against formalin induced licking and flinching in mice**. AGAP at concentrations ranging from 0.25 to 1.0 mg/kg was administered intravenously 20 min before formalin injection. Morphine was administered at a dose of 2 mg/kg i.p. 30 min before formalin injection. The total time spent on licking and flinching of the affected paw was measured in the first (0–5 min) phase and the second (15–30 min) phase after intraplantar injection of formalin. AGAP dose-dependently attenuated the formalin-induced licking and flinching responses in both two phases. ^###^*p* < 0.001 compared with control group; ^∗^*p* < 0.05, ^∗∗∗^*p* < 0.001 compared with formalin group. Data are shown as the mean ± SEM (*n* = 12 per group). *P* values are from two-way ANOVA followed by Bonferroni test.

To further determine whether AGAP induces analgesia effect, we examined paw licking and flinching responses to intraplantar injection of 5% formalin into the hind paw after an injection of AGAP. Formalin concentrations >0.5% induces a biphasic response in rodents (Shields et al., 2010). AGAP resulted in a significant dose-dependent suppression of pain behaviors relative to vehicle-injected mice in both characteristic first (0–5 min) and second (15–30 min) phases of the behavioral response to formalin (**Figure 3**). The inhibition rate of 1.0 mg/kg AGAP were 78.2 and 81.5% for the first phase and second phase of formalin response, respectively (*P* < 0.001, *n* = 12 in each group). The results demonstrated that the suppressive effects of AGAP were similar efficient on both phases. In order to compare the antinociceptive potency of AGAP, another group of mice ware injected with morphine. Similar to AGAP, morphine significantly inhibited pain responses of both phases after formalin injection. The analgesic effect of 1.0 mg/kg AGAP was stronger than 2.0 mg/kg morphine (**Figure 3**, *P* < 0.01, *n* = 12 in each group).

### Rotarod Test

There were no significant differences between control group and 1.0 mg/kg AGAP. The fall-off latencies ranged from 108.64 ± 3.98 to 101.51 ± 4.12 after administration of 1000 nM AGAP (*p* > 0.05, *P* values are from unpaired *t*-test, *n* = 10). The results indicated that AGAP treatments didn’t impair motor function.

### Expression Profiles of Na_V_1.8 and Na_V_1.9 in DRG Neurons

**FIGURE 4:**
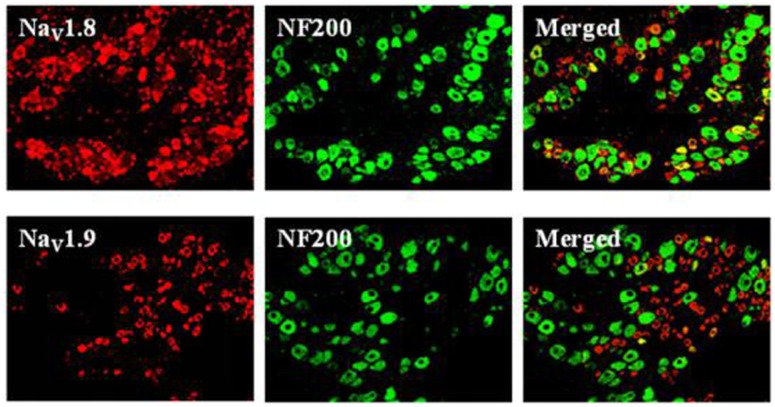
**Expression profiles of Na_V_1.8 and Na_V_1.9 in DRG neurons (×200)**. Double immunofluorescent labeling of L_4-6_ DRG neurons by anti-Na_V_1.8 (red) and anti-NF200 (green) or anti-Na_V_1.9 (red) and anti-NF200 (green) antibodies.

In mammals, acute pain is transmitted to the CNS mainly by two VGSCs, TTX-S Nav1.7 and TTX-R Nav1.8, which are expressed in small-diameter DRG neurons (Dib-Hajj et al., 2010). Nociceptors also express Nav1.9, a second TTX-R sodium current involved in diabetic neuropathy and inflammatory pain (Cummins et al., 1999; Ebersberger et al., 2011). We compared Na_V_1.8 and Na_V_1.9 localizations by double immunofluorescent labeling with NF200, a marker of large- and medium-sized neurons (**Figure 4**). Our results showed that Na_V_1.8 mainly expressed in small- and medium-sized DRG neurons that rarely contained NF200, whereas Na_V_1.9 selectively expressed in small-sized DRG neurons that completely did not contain NF200. This finding is similar to preliminary reported results (Yu et al., 2011; Li et al., 2015). Therefore, DRG neurons with diameters of 15–25 μm (small-sized) were selected for continued experiments.

### Effects of AGAP on Voltage-Dependent Sodium Currents in Rat Amall-Diameter DRG Neurons

**FIGURE 5:**
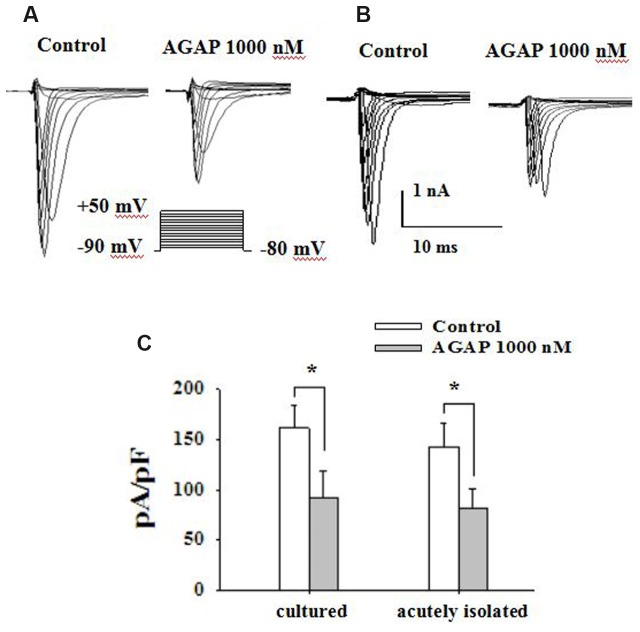
**AGAP inhibits voltage-gated sodium current in primary cultured and acutely isolated small-diameter neurons of rat**. **(A,B)** Following the blockade of calcium and potassium channels, depolarizing voltage commands from -80 to +50 mV from a holding potential of -90 mV elicited fast inward currents. The inward current responses were significantly reduced after the application of AGAP (1000 nM) in cultured and acutely isolated small-diameter DRG neurons. **(C)** The percentage inhibition for *I*_Na_ was 46.9 ± 8.7 and 41.1 ± 6.2% in cultured and acutely isolated small-diameter DRG neurons, respectively (^∗^*p* < 0.05, *P* values are from unpaired *t*-test, *n* = 15 and *n* = 19, respectively).

In electrophysiological testing experiment, voltage-dependent calcium and potassium channels were pharmacologically blocked. Sodium currents were maximally activated by a test pulse at -10 and 0 mV, and peak amplitude at these potential were -5262 ± 928 and -4658 ± 1016 pA in cultured small diameter DRG neurons (**Figure 5A**, *n* = 15) and acutely isolated small DRG neurons (**Figure 5B**, *n* = 19), respectively. AGAP (1000 nM) inhibited the sodium currents in cultured and acutely isolated small DRG neurons, as shown in **Figure 5C** (*P* < 0.05, *n* = 19 and *n* = 15, respectively). 5 min after addition of 1000 nM AGAP, the rate of inhibition for *I*_Na_ was 46.9 ± 8.7 and 41.1 ± 6.2% in cultured and acutely isolated small-diameter DRG neurons, respectively. The inhibition effect calculated as the mean reduction of maximum peak amplitude. The results showed that the pharmacological action of AGAP was similar in primary cultured and acutely isolated small DRG neurons. The acutely isolated small diameter DRG neurons were used to accomplish the following experiments.

### Effects of AGAP on Current Density–Voltage Relationship and Concentration – Dependent Inhibition of AGAP on Sodium Currents

**FIGURE 6:**
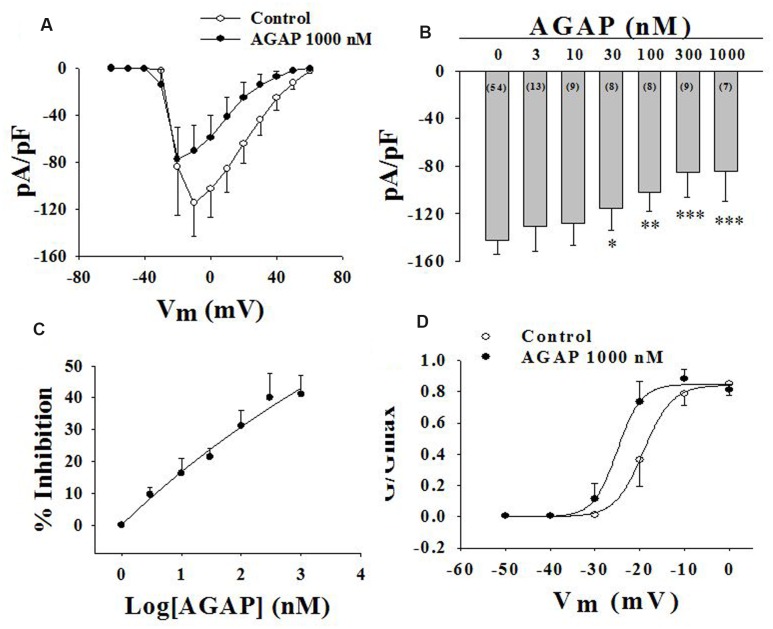
**Effect of AGAP on Na_V_ channel currents in small-diameter DRG neurons**. **(A)** Current density-voltage relationship obtained by plotting current density as a function of test potential under control conditions and after application of 1000 nM AGAP. (*n* = 7). **(B,C)** The graph shows the dose–response relationship of the effect of AGAP in acutely isolated small-diameter DRG neurons (^∗^*P* < 0.05, ^∗∗^*P* < 0.01, ^∗∗∗^*P* < 0.001, *P* values are from unpaired *t*-test. *n* = 8–13). **(D)** Effect of AGAP on steady-state activation kinetics of Na_V_ channels. Data points were fitted with the Boltzmann equation. V_1/2_ was -19.1 ± 0.3 mV under control conditions and -25.0 ± 0.7 mV in the presence 1000 nM of AGAP (*P* < 0.05, *P* values are from unpaired *t*-test. *n* = 7); slope factors were 3.2 ± 0.3 and 2.7 ± 0.3 mV, respectively.

Current density-voltage relationships of sodium currents in small-diameter DRG neurons were shown in **Figure 6A**. AGAP modified the voltage threshold of the activation or the potential at which the inward current reached maximal values. The concentration-responsiveness of AGAP was investigated by measuring current density-voltage relations under different concentrations of AGAP (**Figure 6B**). AGAP (30–1000 nM) reduced the sodium currents in all the DRG neurons. The process that AGAP reduced the inward sodium currents was dose-dependent and the minimal effective concentration was obtained with 30 nM AGAP (*P* < 0.05, *n* = 8). The concentration-responsiveness in acutely isolated rat small-diameter DRG neurons was shown in **Figure 6C**.

Then, we examined the effect of AGAP on activation kinetics of the sodium currents. DRG cells were held at -120 mV, then, the membrane was depolarized from -90 to +60 mV in increments of 10 mV. Boltzmann functions constructed from the average values for *V*_1/2_ and *k* were shown superimposed on the data points with *V*_1/2_ = -19.1 ± 0.3 mV in control and *V*_1/2_ = -25.0 ± 0.7 mV with 1000 nM AGAP in small-diameter DRG neurons (**Figure 6D**, *P* < 0.05*, n* = 7). AGAP shifted the voltage dependency of activation in rat small-diameter DRG neurons. Slope factors were 3.2 ± 0.3 and 2.7 ± 0.3 mV before and after administration of 1000 nM AGAP. Slope factors were not significantly different (**Figure 6D**, *P* > 0.05*, n* = 7).

### Effects of AGAP on TTX-R Sodium Currents in Rat Small-Diameter DRG Neurons

**FIGURE 7:**
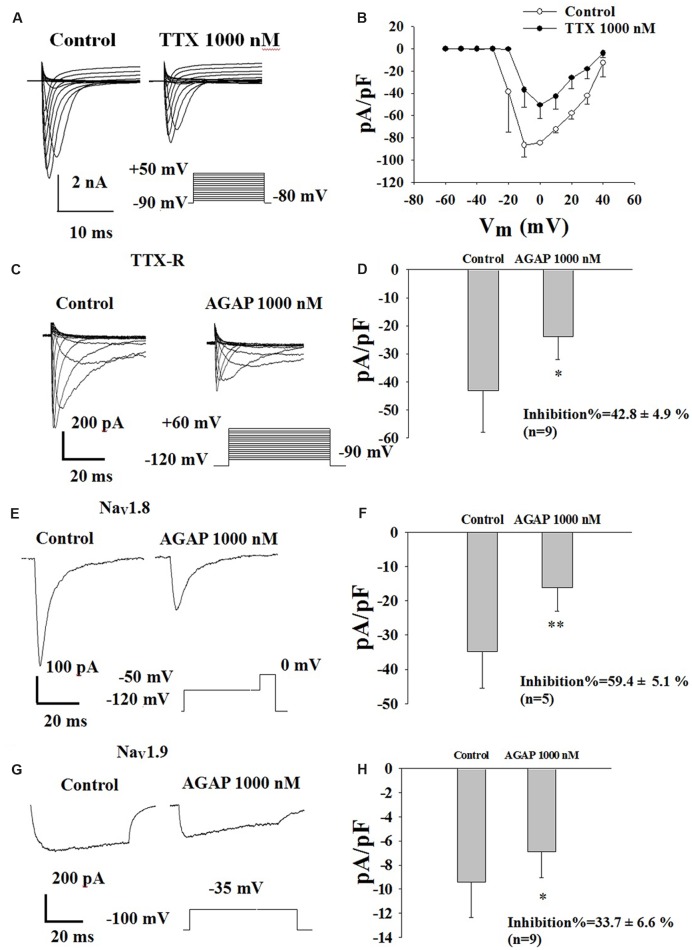
**Effect of AGAP on the TTX-R sodium currents in small-diameter DRG neurons**. **(A)** Representative traces showing the depression of 1000 nM TTX on Na_V_ channel currents in rat small diameter DRG neuron. **(B)** Current density-voltage relationship obtained by plotting current density as a function of test potential under control conditions and after application of 1000 nM TTX (*n* = 6). **(C)** Representative traces showing the depression of AGAP on TTX-R currents in a neuron. **(D)** 1000 nM AGAP inhibited TTX-R currents by 42.8% (*P* < 0.05, *P* values are from unpaired *t*-test, *n* = 9). **(E)** Representative traces showing the depression of AGAP on Na_V_1.8 currents in a neuron. **(F)** At a concentration of 1000 nM, AGAP potently inhibited Na_V_1.8, decreasing current amplitude by 59.4% (*P* < 0.01, *P* values are from unpaired *t*-test. *n* = 5). **(G)** Representative traces showing the depression of AGAP on Na_V_1.9 currents in a neuron. **(H)** 1000 nM AGAP reduced Na_V_1.9 currents by 33.7% (*P* < 0.05, *P* values are from unpaired *t*-test. *n* = 9).

The TTX-S sodium currents of the rat small-diameter DRG neurons could be abolished by 1 μM TTX, indicating that the sodium channels expressing in small-diameter DRG neurons have TTX-S and TTX-R sodium channels (**Figure 7A**). Our current density-voltage relationship of TTX-R sodium currents in DRG neurons was shown in **Figure 7B**. These inward TTX-R sodium currents were maximally activated by a test pulse at -10 and 0 mV (*n* = 6).

In a first series of experiments, calcium, potassium and TTX-S sodium currents were pharmacologically blocked by extracellular application of 4-AP, TEA, La^3+^ and TTX as well as by the addition of Cs^+^ in the recording electrode. Effects of AGAP on TTX-R sodium currents were investigated in DRG neurons after complete inhibition of TTX-S *I*_Na_ currents using 1.0 μM TTX. The results showed that AGAP 1000 nM reduced TTX-R *I*_Na_ peak amplitude by 42.8% (**Figures 7C,D**, *P* < 0.01, *n* = 9). The peak amplitudes were -1070.1 ± 407.9 and -636.2 ± 253.5 nA before and after the application of 1000 nM AGAP.

Effects of AGAP on Na_V_1.8 currents were investigated in acutely isolated rat DRG neurons lacking detectable Na_V_1.9. **Figure 7C** shows representative Na_V_1.8 current traces. Currents were elicited by steps to membrance potential between -90 and +60 mV from a holding potential of -120 mV, at which most channels are in the resting-state. AGAP had obvious inhibitory effects on Na_V_1.8 current amplitudes. 1000 nM of AGAP reduced Na_V_1.8 current amplitudes by 59.4% (**Figures 7E,F**, *P* < 0.01, *n* = 5). Representative Na_V_1.9 current traces are shown in **Figure 7G** in control condition and in the presence of 1000 nM AGAP. Currents were elicited by depolarizing steps to -35 mV from a Vh of -100 mV. AGAP (1000 nM) inhibited Na_V_1.9 current amplitudes at 33.7% (**Figure 7H**, *P* < 0.05, *n* = 9). Mean value of Nav1.8 and Nav1.9 sodium currents reversal potential are +55 and +63 mV, respectively.

### Effects of AGAP on Steady-State Activation and Inactivation Kinetics of Na_V_1.8 Currents

**FIGURE 8:**
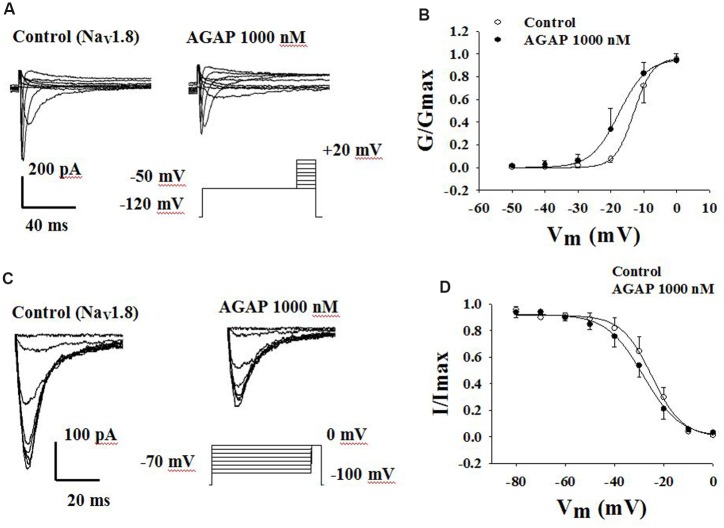
**Effect of AGAP on the dynamic function of Na_V_1.8 channels**. **(A,B)** AGAP shifts the conductance-voltage relationship to more negative potentials. **(A)** Representative steady-state activation of Na_V_1.8 under the control conditions and in the presence of 1000 nM AGAP. **(B)** Normalized activation kinetics determined before and after the application of AGAP. V_1/2_ was -13.0 ± 0.2 mV in control conditions and -17.6 ± 0.4 mV in the presence of 1000 nM AGAP (*P* < 0.05, *n* = 5). Slope factors were 2.8 ± 0.1 and 4.2 ± 0.3 mV, respectively (*P* < 0.05, *n* = 5). *P* values are from unpaired *t*-test. **(C,D)** AGAP had no effect on the voltage-dependence of steady-state inactivation. **(C)** Representative steady-state inactivation of Na_V_1.8 under the control conditions and in the presence of 1000 nM AGAP. **(D)** Normalized peak currents were plotted against membrane potential and Boltzmann equation was used to fit data. *V*_1/2_ was -25.0 ± 0.3 mV in control conditions and -25.4 ± 0.6 mV in the presence of 1000 nM AGAP. Slope factors were -5.5 ± 0.3 and -6.0 ± 0.5 mV, respectively (*P* > 0.05, *P* values are from unpaired *t*-test. *n* = 5).

Voltage-dependent steady-state activation and inactivation properties of sodium channels contribute to membrane excitability. Therefore, some kinetic properties of the Na_V_1.8 and Na_V_1.9 channels were investigated in small-diameter DRG neurons. Na_V_1.8 currents were evoked to -50 mV over the course of 500 ms, followed by the application of voltage steps ranging from -50 to +50 mV in increments of 10 mV (**Figure 8A**). The normalized peak conductance was plotted against voltage and fitted with the Boltzmann equation. AGAP shifted negatively the *V*_1/2_ of activation by 4.6 mV from -13.0 ± 0.2 mV in control conditions to -17.6 ± 0.4 mV (**Figure 8B**, *P* < 0.05, *n* = 5). Slope factors were 2.8 ± 0.1 and 4.2 ± 0.3 mV before and after application of 1000 nM AGAP (**Figure 8B**, *P* < 0.05, *n* = 5).

To study the properties of steady-state inactivation of Na_V_1.8 channels, a series of prepulses from -70 to 0 mV were followed by a testing pulse of 0 mV. AGAP (1000 nM) slightly shifted the inactivation curves of Nav1.8 but there were no significant differences (**Figure 8C**). *V*_1/2_ values were -24.9 ± 0.6 and -28.4 ± 0.6 mV in the absence or presence of AGAP. Slope factors were -6.1 ± 0.5 and -7.4 ± 0.5 mV, respectively. Steady-state inactivations and slope factors were not statistically different in the absence or presence of AGAP (**Figure 8D**, *P* > 0.05, *n* = 5).

### Effects of AGAP on Steady-State Activation and Inactivation Kinetics of Na_V_1.9 Currents

**FIGURE 9:**
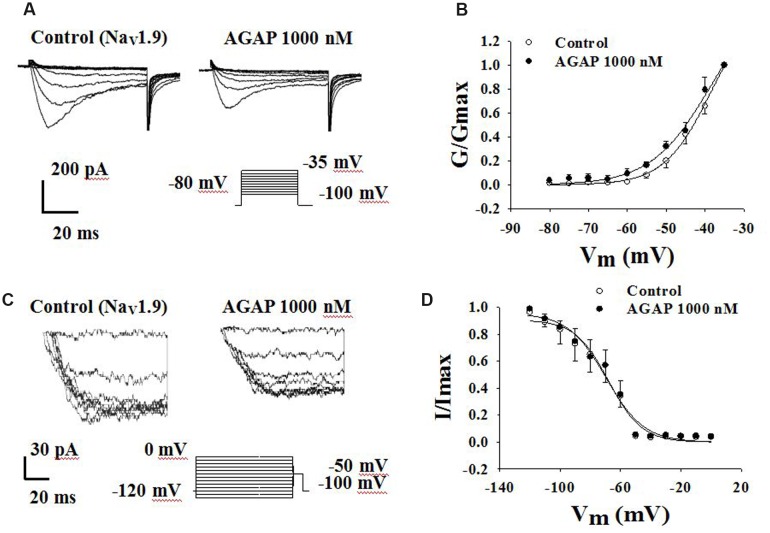
**Effect of AGAP on the dynamic function of Na_V_1.9 channels**. 1000 nM AGAP did not significantly affect the voltage-dependence of channel activation or steady-state inactivation. **(A,B)** Effect of AGAP on steady-state activation kinetics of Na_V_1.9 currents. *V*_1/2_ was -38.4 ± 1.5 mV in control conditions and -38.2 ± 3.7 mV in the presence of 1000 nM AGAP. Slope factors were 6.1 ± 0.5 and 7.8 ± 1.1 mV, respectively (*n* = 7). **(C,D)** Steady-state inactivated of Nav1.9 in the absence and presence of AGAP. *V*_1/2_ for inactivation was -67.9 ± 2.2 mV in control conditions and -69.6 ± 2.5 mV in the presence of 1000 nM AGAP. Slope factors were -11.0 ± 1.8 and -12.5 ± 2.0 mV, respectively (*P* > 0.05, *P* values are from unpaired *t*-test. *n* = 6).

Another TTX-R sodium channel expressed in DRG neurons is Na_V_1.9. Na_V_1.9 generates a low-threshold sodium current with slow rates of activation and inactivation, giving rise to a persistent current component. Fluoride-containing patch pipette solution was used to record Na_V_1.9 currents. Activation properties were studied by applying voltage steps from -80 to -35 mV from a holding potential of -100 mV (**Figure 9A**). AGAP (1000 nM) had no significant effects on the voltage-dependence of the activation curves. *V*_1/2_ for activation was -38.4 ± 1.5 mV in control conditions and -38.2 ± 3.7 mV in the presence of 1000 nM AGAP. Slope factors were 6.1 ± 0.5 and 7.8 ± 1.1 mV, respectively (**Figure 9B**, *P* > 0.05, *n* = 7).

To study the properties of steady-state inactivation of Na_V_1.9 channels, a series of prepulses from -120 to 0 mV were followed by a testing pulse of -50 mV (**Figure 9C**). AGAP (1000 nM) had no significant effects on Nav1.9 channel inactivation (**Figure 9D**). The values of the parameters of *V*_1/2_ and *k* were -67.9 ± 2.2 and -11.0 ± 1.8 mV under control conditions and -69.6 ± 2.5 and -12.5 ± 2.0 mV in the presence of 1000 nM AGAP, respectively (**Figure 9D**, *P* > 0.05, *n* = 6).

## Discussion

Recent evidences that sodium channel activities and expressions altered in peripheral neurons can contribute to the pathophysiology of peripheral neuropathy (Lai et al., 2002; Amir et al., 2006; Dib-Hajj et al., 2010). Scorpion venom has been proved to be a rich source of ion channel regulators. Here, we examined the effect of AGAP, a long-chain scorpion peptides, to anti-nociception by studying behavioral phenotypes of mice. Subsequently, the effect of AGAP on VGSCs was detected in rat small-diameter DRG neurons by whole-cell patch clamp technique.

The results showed that AGAP significantly inhibited the response of writhing in a dose-dependent manner on acetic-acid-induced writhing. We further investigated the antinociceptive role of AGAP in an acute inflammatory pain condition induced by intraplantar injection of formalin. Our results confirmed that AGAP had a significant analgesic activity in both the first and second phases. Besides, AGAP also showed a significant increase in latency to the thermal stimuli in hot plate test, suggests a better antinociceptive activity of AGAP.

Some small peptide inhibitors of N-type channel have shown effect in the treatment of pain. Such as the ω-conotoxin MVIIA has been isolated from venom of Conus magus has been used to treat intractable pain as an analgesic drug (Staats et al., 2004; Stix, 2005). The possible reason is that these inhibitors block the synaptic transmission of signal pathways of pain to the central nervous system (Feng et al., 2001; Yarotskyy and Elmslie, 2009). Most of the long-chain scorpion peptides are the modulators of sodium and calcium channels (Possani et al., 2000; Li et al., 2004). The previous study has shown that AGAP potently inhibited voltage-gated calcium channels in the small diameter DRG neurons (Liu et al., 2014). We have demonstrated that AGAP decreases the HVA calcium channels, especially N-type calcium currents, while N-type calcium channels are expressed in presynaptic and may have more influence on EPSC/EPSP than other calcium type (Tsuzuki et al., 2004). Voltage-gated L-type calcium channels help to maintain longer-lasting depolarizations to reduce firing threshold and regulate repetitive firing as well as shaping regenerative action potentials. Our results strongly suggest that the modulation of VGCCs is a possible mechanism for reduction of neuronal excitability.

These results suggest that antinociceptive effect of AGAP may be ascribed to its specific modulation of voltage-gated ion channels of sensory neurons. Comparing the important role of voltage-gated calcium channels, VGSCs are present in excitable membranes and play a fundamental role in action potentials generation (Payandeh et al., 2011). VGSCs are the important targets for analgesic drugs (Sleeper et al., 2000; Maingret et al., 2008). Next, to determine the effect whether AGAP modulates sodium channels underlying the generation of pain. We found AGAP decreased the sodium currents in a concentration-dependent manner. To our studies, this is the first experimental evidence demonstrating that AGAP is an ion channel regulator with many different actions on a variety of neuronal ion channels including HVA, LVA calcium channels and TTX-R sodium channels.

Nav1.8 and Nav1.9 contain a structural common motif of TTX-R sodium channel subtype that both of them are remarkably expressed in peripheral sensory neurons (Dib-Hajj et al., 2010; Yu et al., 2011), and are thought to play an important role in ectopic discharge in neuronal bodies and axons following peripheral nerve injury (Akopian et al., 1996; Dib-Hajj et al., 1998; Kral et al., 1998; Berta et al., 2008); these properties have identified them as potential molecular targets for analgesic drugs. Both TTX-R subtypes, Nav1.8 and Nav1.9, have been implicated in nociception, including neuronal pain signaling triggered by inflammation (Lai et al., 2002, 2004), and the Nav1.8 is essential for neuropathic pain at low temperatures (Zimmermann et al., 2007). The result confirms that AGAP might attenuate pain by blocking TTX-R channels in small-diameter DRG neurons. Our further results showed that 1000 nM AGAP reduced the Nav1.8 and Nav1.9 currents, the inhibitory percentage of Nav1.8 and Nav1.9 currents were 59.4 ± 5.1 and 33.7 ± 6.6%, respectively. The results suggested that the effect of AGAP on Nav1.8 channels was stronger than that of Nav1.9 channels.

The voltage-gated Nav1.8 channel has been demonstrated having a significantly higher inactivation threshold, slower inactivation kinetics, and a faster recovery from inactivation comparing with TTX-S Na^+^ channels (Cummins and Waxman, 1997), this suggests that Nav1.8 channel is a major contributor to the action potential upstroke in C-type small DRG neurons (Renganathan et al., 2001). The Nav1.8 expression has been reported to increase in the setting of persistent inflammatory pain (Gaida et al., 2005; Villarreal et al., 2005). Some studies suggest that Nav1.8 mutations contribute to pain in some peripheral neuropathies (Faber et al., 2012). Our results showed that AGAP suppresses Nav1.8 currents, suggesting the upstroke of the action potential is blocked by AGAP in sensitive neurons. We predicted that AGAP can reduce Nav1.8 currents, decrease membrane excitability and block AP propagation in nociceptive sensory neurons as well.

AGAP significantly shifted the steady-state activation curves of Nav1.8 currents to more negative potentials, and the inactivation curves of Nav1.8 currents were also shifted to negative potentials. The shift of steady-state activation may speed up the activation process of Nav1.8 channel. At the same time AGAP may also expedite the inactivation process, this is, the time to entry into activation is shortened.

Nav1.8 is important in the development and/or maintenance of nerve injury-induced pain, several Nav1.8-selective inhibitors have been reported to be analgesic in several neuropathic (Jarvis et al., 2007), and it has also been suggested that Nav1.8 might be a potential target of AGAP in inflammatory and neuropathic antinociceptive mechanism.

The Nav1.9 channel is preferentially expressed in nociceptive DRGs and trigeminal nerve neurons (Amaya et al., 2000; Eriksson et al., 2005; Fang et al., 2006), and is known to contribute to the persistent thermosensitive and spontaneous pain by peripheral administration of inflammatory mediators (Crill, 1996; Coste et al., 2004; Priest et al., 2005). The present results showed that AGAP efficiently inhibited Nav1.9 channels in small-diameter DRG neurons in a concentration-dependent manner. The decrease of Nav1.9 currents was associated with subthreshold regenerative depolarization, active hyperpolarizing responses, oscillatory bursting discharges, plateau potentials and bistable membranes behaviors (Maingret et al., 2008). Our results suggested that following the inhibition of AGAP in Nav1.9 channels, the currents reduction of Nav1.9 and the subsequent loss of this depolarizing influence would cause a hyperpolarizing shift in resting potential, elevating the threshold for initiating action potentials.

Nav1.9 channels play a major role in mediating inflammatory rather than neuropathic pain, and contribute to the hyperexcitability of nociceptors observed during inflammatory pain (Priest et al., 2005; Amaya et al., 2006; Ostman et al., 2008). Cummins and Waxman (1997) suggested that, following axotomy, downregulation of Nav1.9 and the subsequent loss of this depolarizing influence would cause a hyperpolarizing shift in resting potential and remove resting inactivation on TTX-S Na^+^ channels, thereby producing hyperexcitability. A link between the Nav1.9-associated persistent current and the pain sensation was demonstrated in neurons from Nav1.9 knockout mice that lack persistent Na^+^ current and have greatly reduced inflammatory hyperalgesia (Priest et al., 2005; Amaya et al., 2006). AGAP reduced the Nav1.9 current, but activation or steady-state inactivation of Nav1.9 was not significantly changed in small-diameter DRG neurons. The results suggest that the AGAP may decrease the effect of Nav1.9 about depolarization of the resting membrane potential, lowering the threshold level for initiating subsequent action potentials (Cummins et al., 1999; Herzog et al., 2001; Baker et al., 2003). AGAP might attenuate inflammatory pain by blocking Nav1.9 sodium channels in nociceptors.

Some researches indicted that TTX-R sodium channels might not be related to the conduction of potential, but have an import role in the action potential generation. The currents reduced by AGAP of Nav1.8 and Nav1.9 channels may also reduce the generation of action potentials. Neurotoxins alter ion channel functions by binding to different sites (Ekberg et al., 2008). Scorpion toxins were individed into α- and β-scorpion toxins. The α-scorpion toxins, which bind to site 3 on VGSCs, slow inactivation processes, and the β-scorpion toxins, which binds to site 4 on VGSC, enhance the activation process. Scorpion depressant β-toxins are considered as analgesic peptides (Chen et al., 2006). According to electrophysiological characteristics of AGAP compared to those of other scorpion toxins, AGAP can be classified as a β-neurotoxin.

In summary, our results have shown that AGAP had a significant anti-nociceptive activity in an animal model of pain. AGAP as potential analgesics is still in the preclinical stage. Although AGAP could modulate the function of calcium channels and TTX-R sodium channels in DRG neurons, AGAP-induced modulation on other ion channels still need to be further investigated. But according to our results, AGAP modulated currents of calcium channels and TTX-R sodium currents in small-diameter DRG neurons, which probably partly contributes to the mechanism of the decreased neuron excitability, blocking neuronal signaling and inducing analgesia.

## Author Contributions

C-FW is the leader of our lab and project. C-LL selected, designed, and wrote the paper. Our work was mainly carried out by X-FL and G-XL and also partly designed by them two. Molecular biological parts was carried out by J-ZC and M-qB. Our peptide, AGAP, was provided from J-HZ and biological support was from Associate YC.

## Conflict of Interest Statement

The authors declare that the research was conducted in the absence of any commercial or financial relationships that could be construed as a potential conflict of interest.
